# Wireless Monitoring of Automobile Tires for Intelligent Tires

**DOI:** 10.3390/s8128123

**Published:** 2008-12-09

**Authors:** Ryosuke Matsuzaki, Akira Todoroki

**Affiliations:** 1 Department of Mechanical Sciences and Engineering, Tokyo Institute of Technology / I1-66, 2-12-1 O-okayama, Meguro-ku, Tokyo 152-8552, Japan; 2 Department of Mechanical Sciences and Engineering, Tokyo Institute of Technology / I1-58, 2-12-1 O-okayama, Meguro-ku, Tokyo 152-8552, Japan; E-Mail: atodorok@ginza.mes.titech.ac.jp

**Keywords:** Intelligent tire, Wireless monitoring, Automobile tire, TPMS, Energy harvesting, Passive monitoring

## Abstract

This review discusses key technologies of intelligent tires focusing on sensors and wireless data transmission. Intelligent automobile tires, which monitor their pressure, deformation, wheel loading, friction, or tread wear, are expected to improve the reliability of tires and tire control systems. However, in installing sensors in a tire, many problems have to be considered, such as compatibility of the sensors with tire rubber, wireless transmission, and battery installments. As regards sensing, this review discusses indirect methods using existing sensors, such as that for wheel speed, and direct methods, such as surface acoustic wave sensors and piezoelectric sensors. For wireless transmission, passive wireless methods and energy harvesting are also discussed.

## 1. Introduction

Intelligent tires, also known as smart tires, are equipped with sensors for monitoring quantities such as air pressure, applied strain, temperature, acceleration, wheel loading, friction, and tread wear, and are expected to improve the reliability of tires and tire control systems such as anti-lock braking systems (ABS). The stimulus for increased research into intelligent tires is attributed to the Bridgestone/Firestone recalls in 2000 [1]. As a result of the recalls, United States Transportation Recall Enhancement, Accountability, and Documentation (TREAD) legislation has mandated that every new automobile be equipped with a tire pressure monitoring system (TPMS) [2-8]. A TPMS employs pressure or other sensor types plus a reliable method for transferring data from inside a pneumatic tire to alert drivers when tires are under-inflated [9-18]. This legislation has given impetus to the development of advanced tire technologies for improved tire safety.

Various reports [19-20] clearly show that adverse road conditions and tire defects play a major role in road traffic accidents. As a consequence, there is urgent need, from a traffic safety point-of-view, for intelligent tires with a warning system for road conditions, optimizing control on poor surfaces, and a tire defect detection system that measures tire deformation, in addition to a TPMS.

The key technologies for the intelligent tires are sensors and data transmission methods. In the case of installing sensors to measure strain applied to a tire, many problems have to be considered. First, because the stiffness of the tire rubber is very low, the conventional foil strain gages designed for metal or plastic materials are not suitable. The high stiffness difference may cause debonding of sensors from tire rubber or degrading performance of the tire because sensors themselves inhibit the deformation of the tire. Second, it is spatially impossible that large-sized sensors are installed in a special environment like the interior space of the tire. Moreover, it is economically difficult to use expensive sensors because tires are comparatively inexpensive products.

In terms of the data transmission, when sensors are installed inside the tire, wireless monitoring is indispensable. Although a slip ring can be used in measurements for rotating objects in laboratory testing, abrasion of the brush and rings may occur and the slip ring does not have a long operational life. Moreover, to activate the sensor, it is necessary to install a battery in the tire. The battery, however, has a limited life and it is difficult for tire users to replace the battery inside the tire. Therefore, energy harvesting or energy scavenging that converts mechanical vibration of tires to electric power has been researched to eliminate the need for battery replacement in recent years. Passive wireless sensors that do not require batteries have also been researched. The passive wireless sensor omits batteries and an energy harvesting system and thus downsizes the instruments installed in the tire. The sensor also has the advantages of decreasing fuel consumption and off-balance problems of the tire due to sensor installation.

This review therefore discusses two key technologies of intelligent tires: 1) tire sensing technology that involves a TPMS and is used in developing an advanced intelligent tire with a tire deformation, wheel loading, or friction measurement system and 2) a system for wireless data transmission between tires and a vehicle that involves active and passive wireless methods and energy harvesting.

## 2. Tire pressure monitoring systems

### 2.1. Indirect pressure monitoring

A simple TPMS method is based on indirect measurements and fuses information from several different physical sensors to compute tire pressure. Persson *et al.* [9, 21] proposed an indirect TPMS using wheel-speed sensors and an electronic control unit (ECU) of ABSs based on vibration and wheel radius analysis. Kojima *et al.* [22] also developed an indirect TPMS using the signal from wheel speed sensors and focusing on the relationship between tire pressure and tire torsional stiffness.

Although indirect systems use existing sensors and are easy to install, the degree of accuracy is not reliable. In particular, changes in road conditions affect indirectly calculated pressure. The combined pressure loss of more than two tires is also problematic. A calibration is often required when one or more tires are changed, or when the pressure is adjusted [16].

### 2.2. Direct pressure monitoring

TPMSs using direct measurements have been developed by many companies such as SmarTire System Inc. of Canada, whose system used clamp-on rim sensors, and Schrader Electronics Ltd. of the United Kingdom, who used valve-attached sensors. The clamp-on rim sensors are fixed on the well bed of the rim with a stainless steel clamp and this fixing method can be applied as an after-sales service. Bridgestone Corp. and Alps Electric Co., Ltd. developed valve-attached sensors, for which the sensor casing is attached to the bottom end of the tire valve.

Conventional capacitive pressure sensors measure capacitance between two electrodes, which changes owing to applied pressure [23]. As the dielectric material for the capacitor, Arshak *et al.* [24] found Nb_2_O_5_ has good sensitivity to applied pressure. Since the sensitivity is related to particle size, small or nanosized particles are good candidate materials for high-sensitivity pressure sensors. This sensor is cost effective and can be produced as a film which is rugged in nature and can operate in many harsh environments.

**Figure 1.**
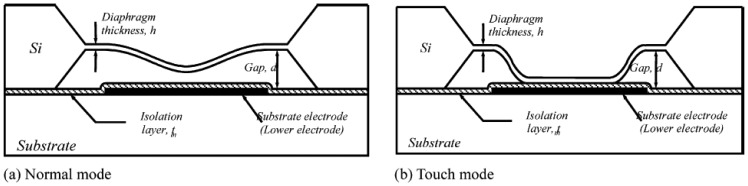
Structure of a touch-mode capacitive pressure sensor: **(a)** normal mode, **(b)** touch mode. The diagram is from Oh *et al.* [25] and is reprinted with permission from Elsevier.

However, the output capacitance is usually nonlinear with respect to input pressure changes and the sensitivity in the near-linear region is not high enough to ignore many stray capacitance effects. To solve this problem, the touch-mode pressure sensors have been developed and are shown in Figure 1 [25-29]. This sensor operates at the instants of two electrodes coming into contact. When two electrodes touch, the contact area increases as the external pressure increases. The advantages of the touch mode operation are good linearity in contact range, mechanical robustness, and large overload protection.

## 3. Advanced intelligent tires

### 3.1. Indirect tire monitoring

A number of advanced tire sensor systems are currently under development. Known as intelligent tires, they are equipped with sensors to monitor quantities such as strain [30, 31], temperature [32, 33], and acceleration [34] in addition to air pressure to improve automobile safety [35-42]. A European Union project, called APOLLO (2002–2005), has been set up for the purpose of developing intelligent tires that can monitor their deformation [20, 43-46]. Tire deformation or strain monitoring enables one to know the amount of friction between the tires and road surface, which can then be used for the optimization of automobile tire control systems, such as the ABS. The use of intelligent tires also benefits other advanced active safety systems, including traction control systems (TCSs), vehicle stability assist (VSA) systems, early detection of tire separation systems [37] and tire-burst prevention systems [47].

There are two types of methods for estimating the friction coefficient: indirect and direct methods. The indirect method does not monitor tire deformation while the direct method does. In most indirect methods, the friction coefficient is determined based on sensing parameters such as the vehicle velocity, wheel angular speed, and normal and tractive forces applied to the tire, axis and wheel [48]. Since the relationships among tire parameters are very nonlinear and complex, quantitative relations are difficult to acquire. Therefore, algorithms such as a fuzzy logic controller [49, 50] or Kalman filter [16, 51-52] are used to estimate tire parameters. Yi *et al.* [53] used the wheel slip, vehicle velocity, and normal load on the tire to determine the friction coefficient and develop a control scheme for emergency braking maneuvers. Miyazaki *et al.* [54-55] measured the four-axis direction force by attaching strain gages to improve the ABS efficiency. Ohori *et al.* [56] and Kamada *et al.* [57] measured the strains applied to the wheel to estimate the six force components in the tire.

These methods require extra sensors such as a strain sensor, yaw rate sensors, acceleration sensors, and steering angle sensors even though they are indirect methods. On the other hand, a reduction in installation cost is possible using only existing sensors. Umeno [58] used the frequency characteristics of the vibration of a wheel rotating at different speeds to estimate tire–road friction. This method requires only wheel speed sensors, which are already used in an ABS. Gustafsson *et al.* [51, 52] proposed a method for estimating the friction between tires and the road surface based on Kalman filtering using existing sensor signals such as individual wheel speeds and an engine torque indicator (injection time or manifold pressure indicator) referred to as a virtual sensor. Bevly *et al.* [59] estimated three key vehicle states—wheel slip, body sideslip angle and tire sideslip angle—using global positioning system (GPS) velocity data. Tire–road friction, effective radius and other tire parameters can also be estimated in real time using a GPS [60-64].

### 3.2. Direct tire monitoring

As opposed to indirect measurements, the direct sensor allows a precise measurement of tire deformation or strain. For direct strain monitoring, a strain gage, based on polyimide film, is the best known and most widely used method. The sensor, however, has a very high degree of stiffness and low elongation compared with tire rubber. This large difference in stiffness may disturb the deformation and stress of tires, and may also cause the debonding of sensors and rubber over a long period of usage. Sensors with low elongation are also easily damaged by large abrupt deformations.

**Figure 2.**
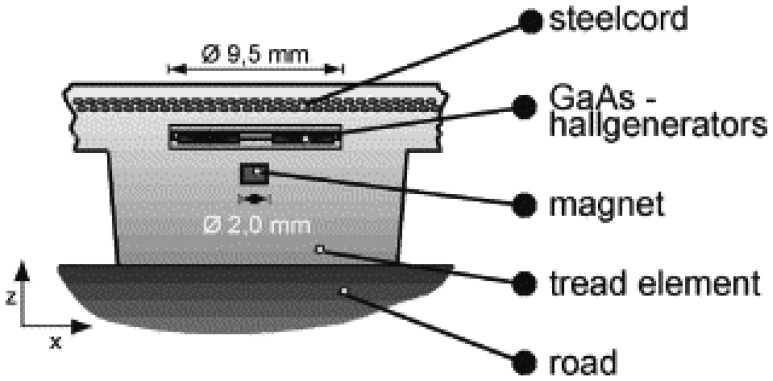
The Darmstadt tire sensor using a Hall sensor for measurements of deformation of a tread element in the tire contact area. The diagram is from Gruber *et al.* [70] and is reprinted with permission from Elsevier.

Surface acoustic wave (SAW) sensors have been proposed for monitoring the deformation during road contact [35, 65-69]. SAW devices use metallic interdigital transducers arranged on the surface of a piezoelectric substrate. SAW devices operate as filters, resonators and delay lines in a growing number of applications. Palmer *et al.* [36] demonstrated the embedment of fiber optic sensors in an automobile tire for monitoring tire strain and captured and measured the onset of a skid. The transducer mechanism is based on an extrinsic Fabry–Perot interferometer (EFPI) using minute changes in the low-finesse Fabry–Perot air gaps. Brandt *et al.* [40, 70] proposed a tread deformation sensor, the Darmstadt tire sensor, which uses GaAs chips glued to thick-film ceramic carriers as shown in Figure 2. The tread element deformation is measured by the sensor as a position change of a magnet relative to four crosswisely arranged Hall sensors. Tjiu *et al.* [71] used microelectromechanical system (MEMS) sensors, including a pressure sensor, accelerometer and temperature sensor for a tire condition monitoring system. Yi [72] used polyvinylidene fluoride (PVDF)-based sensors to measure the tread deformation. Two PVDF deformation sensors are attached on the inner surface of the rubber tire. Savaresi *et al.* [73] embedded piezoresistive low-mass accelerometers mounted inside the tire, which measure the in-tire radial acceleration; the accelerometers are installed in the front-left and rear-left tires.

All these sensors, however, are made of high stiffness materials. Until now, flexible sensors have been developed based on thin technology. Although their flexural rigidity is low, allowing them to be bent, their tensile stiffness is high and elongation low [74, 75]. These sensors are only suitable for very short service periods and, therefore, more reliable sensors with sufficiently low stiffness and high elongation need to be developed.

**Figure 3.**
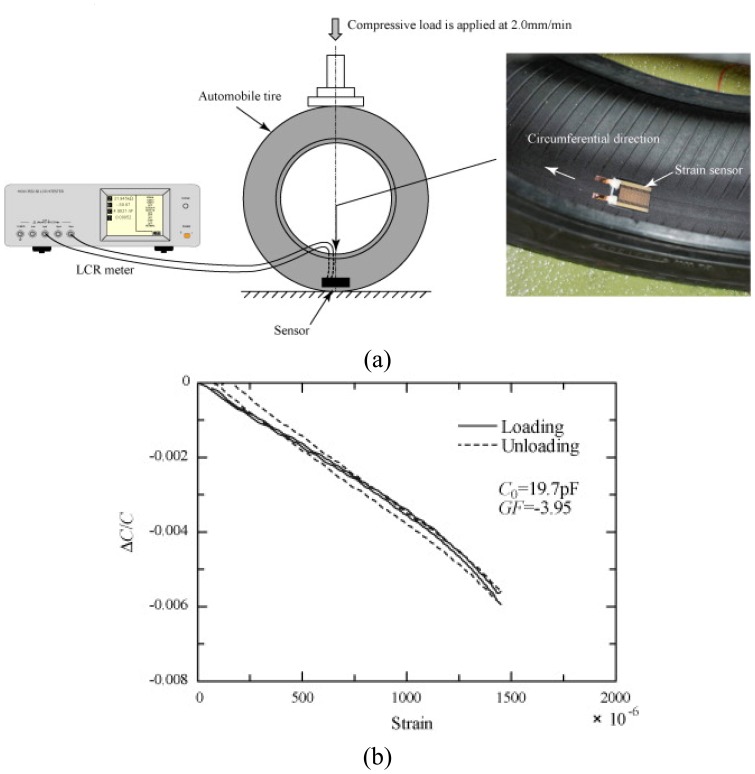
**(a)** Rubber-based sensor attached to the inner surface of a radial tire and **(b)** sensor capacitance change when the tire deforms. The diagram is from Matsuzaki [77] and is reprinted with permission from Elsevier.

Matsuzaki *et al.* [76] proposed a flexible patch-type strain sensor made from flexible polyimide substrates and ultraflexible epoxy resin, which makes the sensor low in stiffness and high in elongation as a whole structure. Matsuzaki *et al.* [77] also proposed a rubber-based strain sensor fabricated using photolithography. The rubber base has the same mechanical properties as the tire surface; thereby the sensor does not interfere with the tire deformation and can accurately monitor the behavior of the tire using sensor capacitance change as shown in Figure 3.

As a non-contact sensing technique for tire rubber, Magori *et al.* [31] presented an ultrasonic tire sensor mounted on the base of the wheel rim inside the tire. The sensor measures continuously the distance to the opposite inner wall of the tire providing highly significant information about the status of the tire. Since the sensor is not in contact with the tire rubber, the inconvenience associated with tire replacement is avoided.

**Figure 4.**
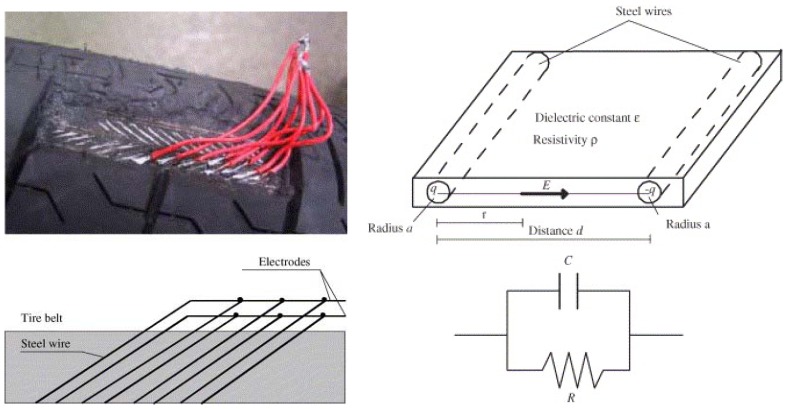
Self-sensor modeling of a steel wire belt in a radial tire as an electric resistor–condenser parallel circuit. The diagram is from Matsuzaki *et al.* [79] and is reprinted with permission from Elsevier.

Without attaching sensors, Matsuzaki *et al.* [78-81] presented a self-sensing method using the tire structure itself as a parallel circuit of a capacitor and resistor as shown in Figure 4. Since the actual tire structure acts as a sensor, no additional sensor is required. Therefore, there is no debonding of the sensor, even during prolonged service, because there is no stiffness difference between the sensor and tire rubber. The measurement system could be small, lightweight and capable of withstanding harsh conditions. Moreover, the method allows for a more direct strain measurement than a method that uses a sensor attached to the inner surface does. Sergio *et al.* [30, 82] also developed a strain monitoring method that adopts the tire itself as a sensing element. The embedded grid of steel wires is used as the electrodes of a distributed array of passive impedances.

## 4. Wireless data transmission

### 4.1 Active wireless transmission

Simple wireless data transmission uses the resonance of a capacitor and an inductor. Data can be converted to the resonance frequency or Q value. To enhance the function of data transmission, most wireless transmission uses an integrated circuit wireless transmitter. Most cases take advantage of the unlicensed ISM (industrial, scientific, and medical) bands, and use communication protocols such as Bluetooth, ZigBee, and IEEE 802.11 [20]. Basically, these wireless communications require a power supply to send a radio signal.

Yi [72] developed piezo-sensor-based intelligent tire system, and used ZigBee wireless communication protocols between a sensor data processing module and a receiving unit. A small battery to power the circuits is located inside the wheel. The receiving unit with an antenna is connected to the onboard laptop. Savaresi *et al.* [73] developed a wireless data transmission of the intire acceleration signal made via a Datatel™ (Langenhagen, Germany) telemetry system. The transmitter and its battery are mounted on the wheel rim. The receiver antenna is placed on the car roof; the acquisition of the sensor signals is made by a DSpace Autobox™ (Michigan, USA) acquisition system. In most cases, wheel-sensor activities are autonomous of any central intelligence and typically require an accelerometer to control wake-up and sleep-mode switching to conserve battery life [83].

Kolle *et al.* [84] developed a low-power sensor for tire pressure monitoring using low-power oscillators. It consists of four tire modules transmitting their data via an HF-link to a central receiver, the hardware of which is shared with the remote keyless entry receiver system. However, a battery limits the operation time of the sensor and wireless communication. To guarantee an effective lifetime of 5–10 years, the battery needs to have a capacity of several hundred mAh, which increases the weight and size of the sensing system.

### 4.2. Energy harvesting

Instead of embedding batteries, mechanical vibration energy can be converted into electricity by capacitive [85], electromagnetic [86-88] and piezoelectric generators [89-90]. Harvesting energy from a rotating tire is a possible method of powering wireless devices implanted in the surface of the vehicle.

Meninger *et al.* [85] proposed capacitive generators for converting ambient mechanical vibration into electrical energy using an MEMS variable capacitor. By placing charge on the capacitor plates and then moving the plates apart, mechanical energy can be converted into electrical energy, which can then be stored. The energy increases as more charge is loaded onto the capacitor. However, the capacitive generators need an initial voltage before they can produce power.

Shearwood *et al.* [86] proposed an electromagnetic generator that consists of a magnet on a polyimide spring. When the generator is vibrated, there is a net movement between the magnet and housing. This relative displacement generates electrical energy by the interaction of the magnet with a planar pick-up coil.

**Figure 5.**
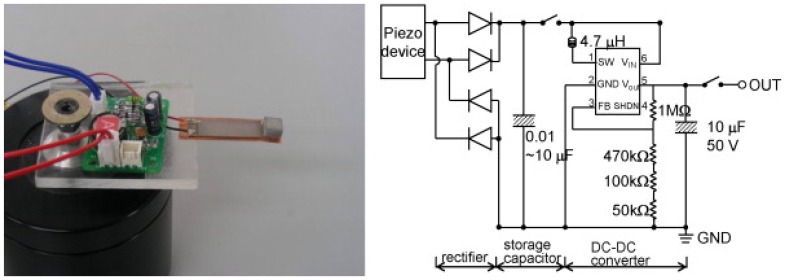
Two-layered piezoelectric bender device for micropower generation. The figure is from Jeong *et al.* [89] and is reprinted with permission from Elsevier.

Elvin *et al.* [91] introduced a self-powered method of sensing and communicating using piezoelectric material. The power is generated solely from the conversion of mechanical strain energy into electrical energy, which is then used to power a wireless link to a receiver and data processing unit. Jeong *et al.* [89] developed bender-type piezoelectric devices for power generator as shown in Figure 5. To match the external vibration frequency with the device resonant frequency, the device consists of two different thick layers, with each layer having different resonant frequency. Snyder [92] also proposed a battery-less TPMS where piezoelectric reeds are included in the tire sensor units and generate electricity.

As an example of other methods, Wang *et al.* [93-94] demonstrated nanowire generators that are driven by an ultrasonic wave to produce continuous direct-current output for harvesting local mechanical energy produced by high-frequency vibration. Qin *et al.* [95] proposed a low-cost approach that converts a low-frequency vibration energy into electricity using piezoelectric zinc oxide nanowires grown radially around textile fibers. By entangling two fibers and brushing the nanowires rooted on them with respect to each other, mechanical energy is converted into electricity owing to a coupled piezoelectric–semiconductor process.

### 4.3. Passive wireless transmission

Although a battery has the advantage of a fixed and stable voltage supply, its disadvantages are its limited total energy available, temperature dependency and relatively short life span. To overcome these problems, a passive wireless system that does not require batteries in the sensor circuit has been developed. A passive wireless system usually involves electromagnetic coupling using two inductors such as a radio-frequency (RF) tag or radio-frequency identification [96, 97]. For passive intelligent tires, Matsuzaki *et al.* [81] used electromagnetic coupling between the two inductors of the antenna and sensor with an inductance capacitance (LC) resonant circuit. Tire deformation changes the sensor's capacitance; then it changes the resonant frequency of the LC circuit. This resonant frequency change is measured as a change in the phase angle for the antenna using electromagnetic induction. Nabipoor *et al.* [23] developed a passive pressure and temperature sensor optimized for a TPMS using an LC circuit and electromagnetic coupling of two inductors. In this passive telemetry LC pressure and temperature sensor, a pressure sensitive capacitor is used in parallel with a temperature sensitive inductor and together they make a LC tank circuit. Changing the applied pressure affects the resonant frequency of the circuit while the temperature affects the bandwidth and amplitude of the impedance at this frequency. However, the energy due to electromagnetic coupling is insufficient to activate the sensor system. This problem also causes the short wireless range.

**Figure 6.**
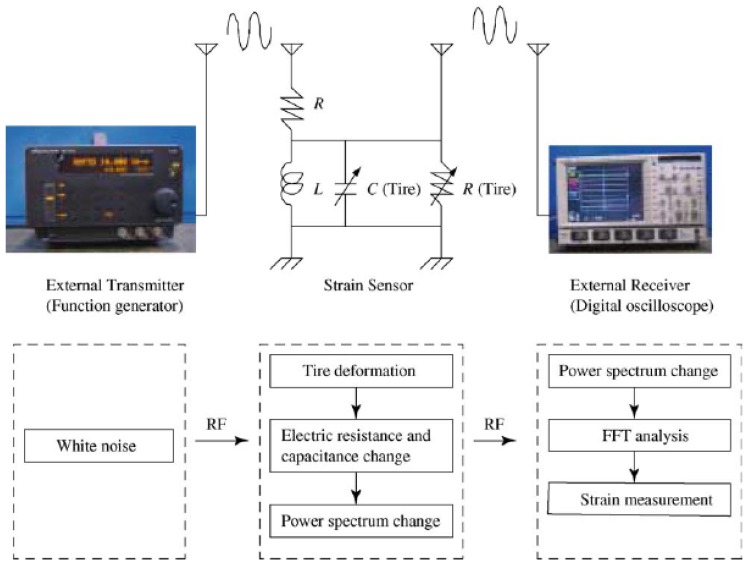
Schematic image of the wireless passive strain measurement system using a tuning circuit and frequency filtering. The diagram is from Matsuzaki *et al.* [79] and is reprinted with permission from Elsevier.

Without magnetic coupling, Matsuzaki *et al.* [79-80] proposed a battery-less sensor using frequency filtering by a tire sensor. The method comprises the sensor or tuning circuit, an external transmitter that emits white noise, and an external receiver as shown in Figure 6. Since the tuning circuit performs as a frequency filter, the tuning frequency of the sensor can be wirelessly measured without any batteries for the sensor circuit. Using spectral features of the tuning frequency and the peak power spectrum and quality factor, tire strain was estimated accurately using a response surface method.

**Figure 7.**
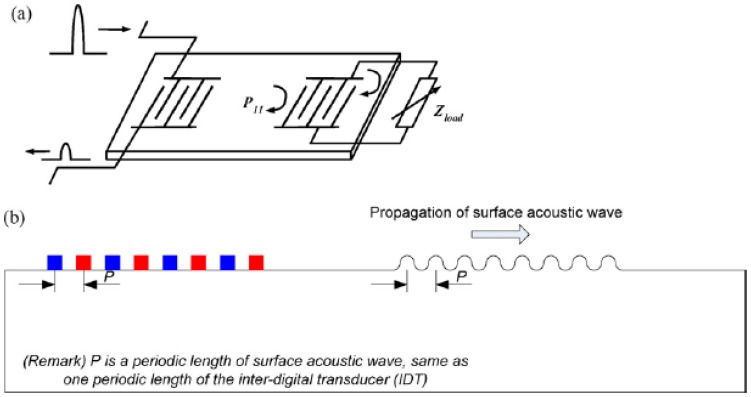
**(a)** Schematic illustration of the SAW traveling wave in piezoelectric substrate and **(b)** a schematic diagram of the traveling wave launched at the SAW inter-digital transducer. The diagram is from Oh *et al.* [25] and is reprinted with permission from Elsevier.

SAW sensors are also powered by the energy of the RF field; thus no battery is required. The SAW device receives incident radio signals and it can reflect them into the air as shown in Figure 7. The reflected signals launching on the surface of the SAW radio transponder contain information such as the impedance and the loaded mass on the substrate. Schimetta *et al.* [33, 66] developed a SAW transponder tag with a capacitive pressure sensor that requires no batteries for a pressure-measurement system. They demonstrated the prototype of a tire pressure sensor unit with a typical accuracy of 15 kPa within a pressure range of 100–400 kPa and an excess pressure stability of 600 kPa. Oh *et al.* [25] also developed a SAW transponder for passive wireless monitoring using the touch-mode pressure sensor. They showed that the maximum distance for detection is about 40 cm and the short range problem when using electromagnetic coupling is solved.

## 5. Conclusions

This review discussed two key technologies required for intelligent tires: sensing and wireless data transmission. Indirect and direct tire sensing technologies for TPMSs and advanced intelligent tires that measure tire deformation for friction estimation were introduced. Although the direct sensing using SAW, fiber optic, piezoelectric, and MEMS sensors have advantages in measurement accuracy compared with the indirect methods, the suitability for tire rubber is problematic. Direct tire monitoring that has better compatibility for tire rubber will be required for long-term service. The wireless transmission between tires and vehicle must be passive because of difficulties in battery installation. A battery-less active system using energy harvesting will be a future technology, and more research is required for gaining sufficient energy.
